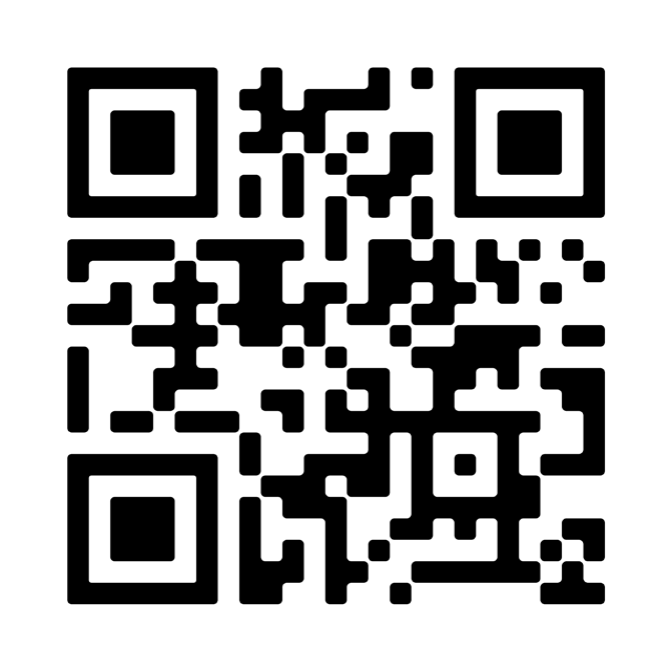# En-block butterfly excision of posterior compartment deep endometriosis: The first experience with the new surgical robot Hugo™ RAS

**DOI:** 10.52054/FVVO.14.5.104

**Published:** 2023-12-13

**Authors:** M Pavone, M Goglia, F Campolo, G Scambia, M.M. Ianieri

**Affiliations:** Dipartimento di Scienze per la Salute della Donna e del Bambino e di Sanità Pubblica, Fondazione Policlinico Universitario A. Gemelli, IRCCS, UOC Ginecologia Oncologica, Rome, Italy; University Hospital Institute (IHU), Institut de Chirurgie Guidée par l’image, University of Strasbourg, Strasbourg, France; IRCAD, Research Institute Against Digestive Cancer (IRCAD) France, Strasbourg, France; Department of Medical Sciences and Translational Medicine, Sant’Andrea University Hospital, Sapienza University of Rome, Rome, Italy; Gynaecology and Breast care center, Mater Olbia Hospital, Olbia, Italy

**Keywords:** Endometriosis, robotic surgery, docking, minimally invasive surgery

## Abstract

**Background:**

Minimally invasive surgery is the gold standard treatment for deep endometriosis when medical management fails. In selected cases, such as when bowel or urinary tract are involved, robotic assisted surgery can be useful due to its characteristics of high dexterity and manoeuvrability. This is the first case of robotic en-bloc excision of posterior compartment deep endometriosis performed with the new Hugo™ RAS system.

**Objective:**

The purpose of this video article is to show for the first time the feasibility of bowel surgery for deep endometriosis with this new robotic device.

**Materials and methods:**

A 24-years-old woman affected by severe dysmenorrhea, chronic pelvic pain, dyschezia and dyspareunia underwent to deep endometriosis excision using the new robotic platform Hugo™ RAS system at the Unit of Gynaecological Oncology, Fondazione Policlinico Universitario A. Gemelli IRCCS, Rome, Italy.

**Main outcome measures:**

Intraoperative data, docking set up, post-operative outcomes up to three months follow up were evaluated.

**Results:**

The surgical procedure was carried out without intra-operative or post-operative complications, operative time (OT) was 200 minutes, while docking time was 8 minutes. No system errors or faults in the robotic arms were registered. Post-operative complete disease-related symptoms relief was reported.

**Conclusion:**

According to our results, the introduction of this new robotic platform in the surgical management of deep endometriosis seems to be feasible, especially in advanced cases. However, further studies are needed to demonstrate the benefits of this surgical system and the advantages of robotic surgery compared to laparoscopy in this subset of patients.

## Learning Objective

Hugo™ RAS is the new robotic platforms introduced for gynaecological procedures by Medtronic. To date, this is the first video on robotically assisted endometriosis surgery performed with this novel system.

## Introduction

Endometriosis is a benign, oestrogen dependent inflammatory disease affecting 10-15% of women of reproductive age (Ianieri et al., 2017). The disease can be localised in the pelvis as peritoneal superficial implants, ovarian endometriomas or deep endometriosis lesions (Giudice, 2010).

Among women with endometriosis, the reported prevalence of rectovaginal or bowel involvement ranges widely, from 5 to 25% (Weed and Ray, 1987). Implants on the bowel proximal to sigma- rectum are frequently associated with general gastrointestinal disturbs as diarrhoea, bloating and abdominal pain (Remorgida et al., 2007). Medical treatment is the first choice in the management of symptoms, but when it fails the surgical excision of the lesions can be resolutive. Although endometriosis is a benign disease, a great surgical expertise is often needed, due to the possible involvement of structures such as bladder, ureters, bowel, and nerves. Moreover, a minimally invasive approach is mandatory, since the majority of patients affected by this pathology are young and in their fertile age. In recent years, we are witnessing a rapid development of robotic technologies, entering a new era of minimally invasive surgical procedures. From its first introduction, 20 years ago, Da Vinci® surgical system (Intuitive Surgical Inc., Sunnyvale, CA, USA), played a crucial role in the advancement of technologies in gynaecologic surgery. Nowadays new platforms are coming out (Peters et al., 2018). In this video article we show for the first time the feasibility of bowel surgery for endometriosis with the new HugoTM RAS system (Medtronic, Minneapolis, USA).

## Patients and methods

A 24-years-old woman affected by severe dysmenorrhea, chronic pelvic pain, dyschezia and dyspareunia underwent to gynaecologic bimanual examination with the evidence of thickening of the uterine torus and utero-sacral ligaments. Pelvic MRI and transvaginal US showed “kissing ovaries”, bilateral endometriomas, a lesion of 25x20 mm at the level of uterine torus extending to the utero- sacral ligaments on both sides, and a thickening of the anterior rectal wall for the presence of a 24x6mm nodule with endometriotic appearance. The patient underwent to hormonal treatment with Dienogest 2 mg for 6 months without evidence of symptoms regression. She was Caucasian, with a body mass index (BMI) of 19.82, nulliparous, with future fertility desire. After signing the informed consent, she underwent a robotically assisted procedure involving deep endometriosis excision. Under general anaesthesia, the patient was placed in lithotomy position with both legs in Allen stirrups, a Trendelenburg tilt and arms along the body. Uterine manipulation aided in the opening of the pouch of Douglas and in the visualisation of the retro-uterine space. Pneumoperitoneum was induced with a trans-umbilical 12 mm robotic optic trocar. The “straight” 8 mm trocar placement and the “compact” robotic docking configuration were set according to the indications of Gueli Aletti et al. (2022). A fourth non-robotic 5 mm trocar was inserted in the Palmer’s point for the assistant’s instrument. Bipolar fenestrated grasper, monopolar curved scissor and needle driver were used for the procedure. The first surgeon position was at the console controlling the movement of instruments and camera. The first assistant was on the patient’s left side. The second assistant, situated between the patient’s legs, was in charge of placing and moving the uterine manipulator. The first surgeon had completed the official technical training delivered by the company before the operation.

## Results

The first surgical inspection of the abdomen and pelvis showed bilateral diaphragmatic domes, cecum, and ileum with no signs of endometriotic lesions. In the pelvis, the uterus appeared regular in size with the ovaries attached to its posterior wall presenting bilateral endometriomas of 2.5-3 cm. The rectum appeared retracted and strongly adhering with the uterine torus and the ovaries, due to the presence of a nodule with an endometriotic appearance. The step-by-step procedures performed included: 1) left and right ovarian cortex incision and cystectomy 2) bilateral ovarian suspension with straight needles 3) left and right medial para-rectal spaces dissection 4) left and right presacral spaces openings with hypogastric nerves visualisation 5) latero-medial recto-vaginal spaces dissection 6) pelvic peritoneum excision 7) uterine torus nodule excision 8) 2 cm of rectal shaving up to the muscular layer without mucosal incision and 3/0 Vicryl suture apposition to reinforce the rectal wall 9) endobag specimens extraction. A drainage was positioned at the end of the procedure to monitor post-operative bleeding. No intra or post-operative complications were reported. No system errors or faults in the robotic arms were registered. Estimated blood loss was 100 cc, docking time of 8 minutes and total operative time 200 min. The patient was discharged on the third postoperative day. Symptoms recurrences were not shown at 1 and 3 months of follow up.

## Discussion

The video highlights, for the first time, the use of a novel platform in the surgical treatment of endometriosis involving the posterior compartment and large bowel. The pre-operative docking and positioning of the trocars allowed access to all important anatomical regions without collision of the robotic arms. Docking time of 8 minutes was in line with the previous published literature data (Raffaelli et al., 2023). The mean operating time is reported to be longer when procedures are performed robotically with respect to laparoscopic ones (Restaino et al., 2020). For this patient the operating time of 200 min was similar to the robotically assisted surgeries reported by other authors (Nezhat and Sirota, 2014; Soto et al., 2017). While there are potential benefits associated with the use of robotic-assisted surgery, the current advantages remain a topic of debate. In the realm of endometriosis surgery, laparoscopy continues to be considered the preferred and most reliable approach (Ercoli et al., 2017). Robotic surgery, on the other hand, offers certain advantages such as the use of articulating instruments that surpass the range of motion of rigid laparoscopic instruments, and a 3D magnification view. These features are particularly beneficial in the complex management of advanced-stage deep endometriosis (Luu and Uy-Kroh, 2017). Furthermore, the estimated blood loss and the hospital stay are shorter when gynaecological procedures are performed by robotic-assisted surgery (Marchand et al., 2021). This novel system commercialised in 2021, exploits the advantages of modular multi-port robots with four independent bedside units able to move around the operating room and the patient’s table. Most of the new robotic platforms are still not on the market and validation studies in the different fields are ongoing to demonstrate the safety and feasibility of these devices. Studies on the application of this robotic device to gynaecological procedures were carried out (Monterossi et al., 2022), and uro-gynaecological surgeries have been described (Panico et al., 2023). To date, this is the first video showing the feasibility of the HugoTM RAS assisted surgery in patients affected by endometriosis involving large bowel. Surgical management of bowel endometriosis depends on the lesion’s depth of infiltration, size, number of anatomic sites involved, presence of multifocal or multicentric lesions as well as the surgeon’s preference. The decision to perform conservative nodule excision by rectal shaving or discoid resection instead of segmental resection is usually taken when the maximum diameter of the lesion does not exceed 3 cm, with maximum involvement of one-half of the bowel circumference (Fanfani et al., 2010). Due to the small size of the nodule in this patient a rectal shaving was performed with a complete excision as a result.

The introduction of this surgical system in a tertiary referral centre for minimally invasive and robotic endometriosis surgery, together with the described docking setup and the surgical results showcased the possibility to successfully incorporate this technology into the surgical management of endometriosis. The outcomes indicated that this approach was safe, and it effectively relieved symptoms associated with the condition.

## Conclusions

According to our results, the introduction of this new robotic platform in the surgical approach of deep endometriosis seems to be feasible, aiding in the surgical management of advanced cases in terms of aesthetic results, length of hospitalisation and resolution of symptoms. Further studies with a larger population are needed to demonstrate the benefit of this surgical system and the advantages of robotic surgery compared to laparoscopy in this subset of patients.

## Video scan (read QR)

https://vimeo.com/manage/videos/842179728/8084ab550c

**Figure:**